# Infarct tissue characteristics of patients with versus without early revascularization for acute myocardial infarction: a contrast enhancement cardiovascular magnetic resonance imaging study

**DOI:** 10.1186/1532-429X-13-S1-P145

**Published:** 2011-02-02

**Authors:** Marlon A Olimulder, Karin Kraaier, Michel A Galjee, Marcoen Scholten, Jan van Es, Lodewijk Wagenaar, Job van der Palen, Clemens von Birgelen

**Affiliations:** 1 MST Enschede, Enschede, Netherlands

## Introduction

Histopathological studies suggested that early revascularization for acute myocardial infarction (MI) limits size, transmural extent, and homogeneity of myocardial necrosis. However, the long-term effect of early revascularization on infarct tissue characteristics is greatly unknown. Cardiovascular magnetic resonance (CMR) imaging with contrast enhancement (CE) allows non-invasive examination of infarct tissue characteristics and left ventricular (LV) dimensions and function in one examination.

## Purpose

In a consecutive series of patients with prior MI in whom CE-CMR was performed for clinical reasons, we compared data from patients with versus without early revascularization for acute MI. Based on the findings of previous histopathological studies, we hypothesized that in patients with early revascularization, infarct areas may be smaller, less homogeneous, and less transmural on CE-CMR than in patients without early revascularization.

## Methods

A total of 86 patients, referred for cardiac evaluation for various clinical reasons, were examined with CE-CMR >1 month (median 11, range 1-425) post-acute MI. We compared patients with (n=44) versus without (n=42) successful early revascularization for acute MI. Cine-CMR measurements included left ventricular (LV) end-diastolic and end-systolic volumes, LV ejection fraction (LVEF,%), and wall motion score index (WMSI). CE images were analyzed for core, peri, and total infarct size (%), and for the number of transmural segments.

## Results

**Figure 1:**
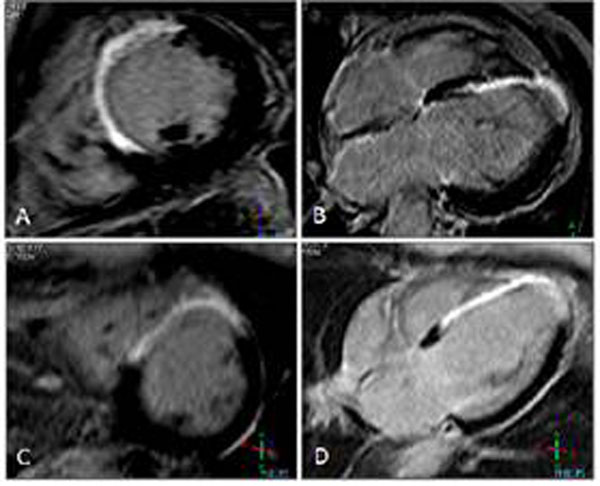
(A,B) CE-CMR imaging short axis and four chamber view in an early revascularized patient, presence of transmural CE is observed anteroseptal and apical. (C,D) CE –CMR imaging short axis view and four chamber view in a patient without early revascularization; presence of transmural CE is observed anteroseptal and apical.

Patients with successful early revascularization had a better LVEF (41±18 vs. 33±14%; P=0.02), a superior WMSI (1.12, range 0.00-2.76 vs. 1.42, range 0.00-3.00; P=0.02), and smaller end-systolic volumes (142±79 vs. 179±94; P=0.05) than patients without early revascularization. However, neither groups showed a difference in core (10±6 vs. 10±6%), peri (10±4 vs. 10±4%), and total infarct size (20±10 vs. 20±9%), or transmural extent (P>0.30 for all). Figure 1.

## Conclusion

Long-term survivors of acute MI with versus without successful early revascularization showed no difference in infarct tissue characteristics as assessed by CE-CMR. Nevertheless, LV

function was significantly lower in patients without early revascularization, who also tended to show other signs of LV remodelling.

